# Sarcoma metastasis: distinct biological principles beyond the carcinoma paradigm

**DOI:** 10.1007/s10555-026-10360-z

**Published:** 2026-07-20

**Authors:** Nadja Chevalier, Paul C. Nathan, Ivan Stamenkovic

**Affiliations:** 1 Division of Pediatric Hematology-Oncology, Department of Pediatrics, Lausanne University Hospital, Lausanne, Switzerland; 2 Division of Hematology and Oncology, The Hospital for Sick Children, University of Toronto, Toronto, Canada; 3 Experimental Pathology Service, Lausanne University Hospital, University of Lausanne, Lausanne, Switzerland

**Keywords:** Sarcoma metastasis, Mesenchymal stromal cells, Metastasis-initiating cells, Tumor microenvironment, Epigenetic plasticity

## Abstract

Sarcomas are rare in adults but constitute a substantial fraction of pediatric malignancies and are characterized by a high propensity for hematogenous dissemination and poor outcomes once metastatic. Yet, most mechanistic understanding of metastasis derives from carcinomas and is frequently extrapolated to sarcomas despite major biological differences. This review synthesizes current evidence on sarcoma metastasis and highlights key knowledge gaps across the metastatic cascade. It frames sarcomas as malignancies often arising from mesenchymal stromal cells, whose intrinsic motility, plasticity, and stem-like features may predispose transformed cells to dissemination without requiring a classic epithelial-to-mesenchymal transition program. The concept of metastasis-initiating cells is discussed in the context of sarcoma cancer stem cell biology, clonal evolution, and epigenetic reprogramming, emphasizing that the relative genomic simplicity of certain pediatric sarcomas driven by recurrent fusion oncogenes may provide powerful models to dissect metastasis dependencies. The review examines how the sarcoma tumor microenvironment, often immunosuppressive and macrophage-rich, may shape invasion, intravasation, survival in circulation, extravasation, and organotropism, particularly the strong predilection for pulmonary metastasis. It summarizes emerging data on circulating tumor cells and complementary liquid biopsy approaches, while underscoring technical limitations caused by sarcoma heterogeneity and lack of robust markers. Finally, the review appraises current treatment limitations for metastatic sarcoma and argues that improved mechanistic resolution, especially of plasticity, epigenetic states, microenvironmental interactions, and dormancy, will be essential to identify actionable vulnerabilities and improve outcomes for patients with disseminated disease.

## Introduction

Scientific literature on cancer metastasis has a heavy emphasis on carcinomas. Comparatively fewer studies have been conducted on sarcoma metastasis. Sarcomas comprise approximately 1% of adult cancers but about 15% of pediatric tumors [1]. These heterogeneous tumors arise in mesenchymal tissues and often display aggressive behavior with high metastatic proclivity [1]. The prognosis of disseminated sarcomas is poor, despite multimodal therapeutic approaches. Currently, over 70 sarcoma subtypes have been classified based on histological and genetic features. Genetic analyses divide sarcomas into two main classes. One consists of tumors displaying marked genetic instability, leading to multiple complex karyotypic abnormalities with no specific pattern. Sarcomas in this class tend to occur in older patients and have a relatively high mutation rate in the p53 and RB signaling pathways. The other class comprises malignancies harboring single, recurrent karyotypic defects, a significant proportion of which are chromosomal translocations, believed to underlie their pathogenesis. Most genes located at the chromosomal breakpoints encode transcription factors and transcriptional regulators [2]. In most cases, the fusion gene generated by the chromosomal translocation encodes an aberrant transcription factor or transcriptional regulator, which, either alone or in combination with other genetic events, drives transformation by altering the gene expression repertoire of permissive cells [1].

Metastasis is the primary cause of cancer mortality and morbidity. The metastatic cascade is a multistage process that includes local migration of and invasion by malignant cells, intravasation into and survival in the vascular or lymphatic circulation, extravasation, colonization of a distant organ, and establishment of a microscopic tumor colony [3]. If the colony survives, it could become a macroscopic secondary tumor. Several factors influence a tumor cell’s propensity to metastasize, including its origin, the properties it has acquired as a result of accumulated genetic and epigenetic modifications, and host tissue features. Multiple mechanisms contribute to metastasis, and they may differ according to tumor type.

Most metastasis research has been done in carcinomas, and the insights gained tend to be extrapolated to sarcomas. However, these two cancer types have different biological, genetic, and behavioral properties, so extrapolations may be inaccurate. Unlike carcinomas, sarcomas do not or rarely disseminate through the lymphatic system — lymph node involvement is uncommon. About a third of sarcomas bear a single major genetic event with a low mutational burden, whereas carcinomas bear multiple complex genomic and genetic defects, making it hard to identify genetic and epigenetic drivers at different stages of tumor progression [4]. Epithelial-to-mesenchymal transition (EMT) is considered a prerequisite for metastasis formation in carcinomas, but EMT probably does not have the same significance in sarcomas because they are mesenchymal in origin [5]. Table 1 summarizes the core principles discussed throughout the review, contrasting carcinoma-derived models of metastasis with sarcoma-specific concepts. This narrative review synthesizes selected experimental, translational, and clinical observations to identify knowledge gaps in sarcoma metastasis and contrast sarcoma and carcinoma metastatic behavior.

**Table 1:** Core principles distinguishing sarcoma metastasis from the carcinoma paradigm

Core principles	Carcinoma paradigm	Sarcoma-specific concept
*Intrinsic dissemination capacity*	Metastasis is commonly framed as the acquisition of mesenchymal traits by epithelial tumor cells through EMT	Sarcoma cells arise from mesenchymal tissues and may therefore already possess intrinsic dissemination capacity as pre-existing mesenchymal competence for motility and invasion. Therefore, classical EMT may be less central, as sarcoma cells may instead modulate pre-existing programs
*Reduced reliance on genetic diversification*	Metastatic progression is often interpreted through progressive acquisition of mutations, clonal diversification, and selection	Variable dependence on progressive point-mutation accumulation, with important contributions from transcriptional, epigenetic, state-dependent, and copy number changes
*Early metastatic competence*	Early dissemination can occur, but whether early disseminated cells are fully metastasis-competent remains context-dependent	In high-grade pediatric sarcomas, micrometastatic disease is often already present at diagnosis, suggesting that dissemination capacity may be acquired early and may partly exploit pre-existing mesenchymal migratory programs
*Immune-poor mesenchymal microenvironment*	Tumor and stromal compartments are usually more clearly separable; immune infiltration can support checkpoint blockade sensitivity in selected tumors	Mesenchymal origin can blur the distinction between tumor and stromal compartments. Low mutational burden and immune-cold or myeloid-rich TME may favor immune escape and limit checkpoint blockade efficacy
*Predominant hematogenous dissemination*	Dissemination is often framed through lymphatic spread to regional lymph nodes, which is central to TNM staging and prognosis, although direct hematogenous dissemination also occurs	Most sarcomas disseminate predominantly through the bloodstream, with lymph node involvement being uncommon except in selected subtypes. This pattern may reflect intrinsic mesenchymal traits together with vascular and lymphatic accessibility
*Lung-predominant organotropism*	Carcinomas exhibit tumor-type-specific organotropism with diverse metastatic patterns (liver, bone, lung, brain) depending on the primary tumor type	Despite sarcoma heterogeneity, lung metastasis predominates across most sarcoma types. This lung tropism remains incompletely explained by passive vascular filtering alone and likely reflects organ-specific survival cues, stromal support, and tumor-intrinsic adaptations

## Sarcoma cells of origin and metastases

### Mesenchymal stromal cell properties

A cancer *cell of origin* is defined as the normal cell that acquires genetic and epigenetic modifications and becomes malignant [6]. The cell of origin of a cancer may play a major role on the fate and behavior of the transformed cells, including their propensity to form metastases [7].

**Fig. 1:**
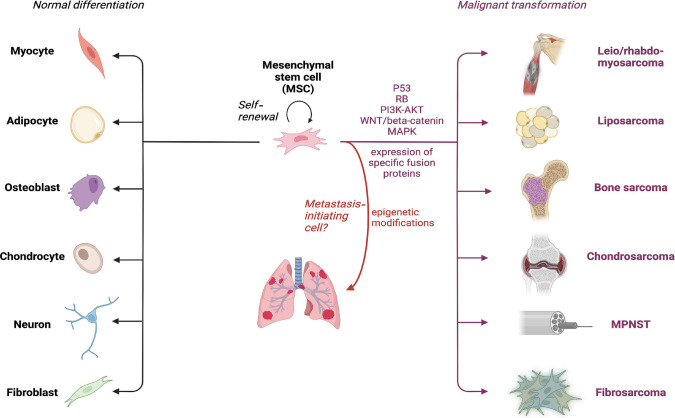
MSCs can differentiate into various cell types that form connective tissues. During various MSC differentiation stages, if certain signaling pathways are deregulated or sarcoma-specific fusion proteins are overexpressed, sarcomagenesis could be triggered. In addition, epigenetic modifications could lead to MSCs inducing metastasis. This figure was created with BioRender

Sarcomas originate in connective tissues. Although they are heterogeneous malignancies with wide-ranging behavioral differences, accumulating evidence suggests that mesenchymal stromal cells (MSCs), also known as mesenchymal stem cells, or closely related mesenchymal progenitors can act as cells of origin or permissive target cells for transformation in several sarcoma subtypes [8]. MSCs can self-renew and differentiate into many cell types, including osteoblasts, adipocytes, myoblasts, chondrocytes, and fibroblasts, which can characterize the phenotype of diverse sarcoma subtypes [9] (Fig. 1). Sarcoma phenotypes may be determined by the differentiation stage of MSCs at which they are permissive for transforming oncogenic events and/or by transcriptomic changes induced by these events. Several studies have shown that, in MSCs, the deregulation of major signaling pathways, including the P53, RB, PI3K-AKT, WNT/beta-catenin, and MAPK pathways, induce malignant transformation and sarcomagenesis. Moreover, MSCs that express sarcoma-specific fusion proteins can form tumors with the defining features of sarcoma subtypes that harbor the same chromosomal rearrangements [10–12].

MSCs may also harbor properties required for metastasis. Cancer stem cells (CSCs) that express MSC markers and retain MSC differentiation properties have been identified in several sarcoma subtypes, including synovial sarcoma, osteosarcoma and chondrosarcoma [12, 13]. In addition to their self-renewal and differentiating abilities, CSCs can initiate tumor growth, although not all tumor-initiating cells (TICs) are CSCs. Two main models that are not mutually exclusive have been proposed to explain tumor origin and heterogeneity. In the clonal evolution model, a normal cell becomes malignant after accumulating genetic and/or epigenetic modifications and gives rise to a clone of neoplastic cells. Additional genetic changes and selection drive tumor heterogeneity [14]. The alternative CSC model suggests that cancer cells are organized hierarchically, where only the cells at the top of the hierarchy possess self-renewal capacity, plasticity, and tumor-forming ability [15]. Although they account for a small percentage of malignant cells, CSCs are frequently enriched in advanced cancers, as demonstrated by the high success rate of patient-derived xenograft formation in clinically aggressive, undifferentiated sarcomas compared to well-differentiated tumors [16]. This observation questions whether metastasis-initiating cells (MICs) are actually CSCs or a derivative thereof.

Strong evidence suggests that, in carcinomas, acquiring a mesenchymal state through EMT is accompanied by acquiring stem-like features [17]. Because sarcomas have a mesenchymal origin, many or all of their cells may bear the kind of plasticity, including stemness features, that renders them prone to metastasize.

### MICs

MICs are the subset of rare tumor cells that retain the capacity to pass through the selective pressures of dissemination and to establish productive growth at a distant site. Across cancer types, two non-mutually exclusive models may help clarify this concept. In the *lineage-defined model*, MICs correspond to a relatively stable cancer stem-like population inherited from, or selected within, a permissive progenitor lineage [18]. In the *state-dependent model*, MIC capacity is not restricted to a fixed cellular hierarchy but reflects a transient and reversible cellular state, shaped by transcriptional, epigenetic, genomic, and microenvironmental pressures [18]. In sarcoma, available experimental data support elements of both models. Using molecular barcoding and lineage tracing, Tang et al. showed that CTCs and early metastases can be polyclonal, whereas advanced sarcoma metastases may derive from a single clone that can be traced back to the primary tumor [19]. Conversely, in Ewing sarcoma, a miR-145-based live reporter assay isolated miR-145^low^ cells with enhanced clonogenicity, tumor-initiating capacity, and metastatic proclivity compared with miR-145^high^ counterparts, consistent with the concept that aggressive behavior may reflect a dynamic cellular state rather than a fixed marker-defined hierarchy [20].

This distinction is relevant in sarcomas because mesenchymal origin and plasticity may blur the boundary between stable cancer stem cell identity and dynamic cell-state adaptation. MICs have therefore been difficult to identify using static markers alone. Analyzing circulating tumor cells (CTCs) for stemness- or plasticity-associated features may help, but remains technically challenging because metastasis is inefficient and only a very small fraction of CTCs successfully seed secondary tumors [21].

Collectively, available studies in sarcoma suggest that metastatic competence may reflect a hybrid model, in which pre-existing subclones or lineage-associated programs are selected during dissemination, while dynamic transcriptional and epigenetic states modulate the ability of individual cells to survive, seed, and colonize distant sites. However, integrated longitudinal studies combining clonal tracking with single-cell transcriptomic, epigenomic, and spatial profiling of matched primary tumors, CTCs or disseminated tumor cells, and metastatic lesions remain limited.

Importantly, sarcoma metastatic evolution should not be assessed only through point mutations. Copy-number alterations (CNAs), including broad chromosomal gains and losses, focal amplifications or deletions, and whole-genome doubling, provide another substrate for metastatic selection. Large-scale genomic studies show that many adult soft-tissue sarcomas, particularly complex-karyotype sarcomas, are characterized by copy-number changes and low mutational burden, although CNAs may also occur as secondary or co-occurring events in fusion-driven sarcomas [22]. Pan-cancer longitudinal analyses further suggest that CNAs may be enriched during metastatic dissemination more than point-mutational accumulation, supporting consideration of structural genomic evolution alongside transcriptional and epigenetic mechanisms [23].

Epigenetic plasticity also helps explain MIC states and metastatic competence. Its contribution to metastasis is increasingly recognized but difficult to dissect, as causal epigenetic drivers must be distinguished from passenger or adaptive changes. In this context, fusion-driven pediatric and young adult sarcomas may represent particularly tractable models, because transformation is often initiated by a dominant fusion oncogene in a relatively low mutational burden background. Several of these fusion proteins act as aberrant transcriptional or chromatin regulators. For example, EWSR1-FLI1 in Ewing sarcoma reshapes enhancer activity, including at GGAA microsatellite-associated enhancers [24], while SS18-SSX in synovial sarcoma alters BAF chromatin-remodeling complex targeting [25]. These examples illustrate how sarcoma drivers can couple oncogenic transformation to epigenetic cell-state regulation. Importantly, this framework does not exclude genomic evolution. Copy-number alterations and other structural genomic changes should be considered complementary contributors to sarcoma metastatic competence, particularly in complex-karyotype sarcomas.

### MIC release from the primary tumor

In carcinomas and sarcomas, MIC release from the primary tumor is an early event and may occur even when the primary tumor is undetectable [26, 27]. However, there is frequently a considerable delay, sometimes decades, between a potential MIC detaching from the primary tumor and a macroscopic metastatic growth appearing. This time interval may partly result from a cellular state termed *dormancy* (see Sect. 8.4).

Before the advent of chemotherapy, 90% of patients with non-metastatic osteosarcoma at diagnosis, who were treated by surgical resection alone, developed metastasis 6 to 36 months later, indicating that most patients with osteosarcoma have micrometastases at diagnosis [28]. In high-grade pediatric sarcomas, micrometastatic disease is more the rule rather than the exception, indicating that the cells with the genomic alterations required for metastasis formation are present when the primary tumor forms; time-dependent accumulation of new mutations is not needed [29]. In physiological conditions, MSCs can migrate through the circulation into injured tissues during tissue repair [30]. This suggests that at least some of the resources required for sarcoma cells to disseminate are already available within their cell of origin. Sarcoma cells may only need to adjust their gene expression repertoire and levels to exploit these resources advantageously.

## Role of the tumor microenvironment in metastasis

The tumor microenvironment (TME) strongly influences metastasis. For example, xenografted cancer cells cannot generate metastases from ectopic sites (e.g., subcutaneous) but induce robust metastasis when injected orthotopically [31]. The TME is composed of extracellular matrix, endothelial cells, immune cells, and stromal cell types, including fibroblasts [32]. However, its primary components may vary between tumor types. In sarcomas, this analysis is complicated by the mesenchymal origin of malignant cells, which makes tumor and non-malignant stromal cells difficult to distinguish because unique markers are often lacking [29]. As a result, the sarcoma TME remains less well characterized than that of carcinomas. Nevertheless, available data suggest that the sarcoma TME is not a passive background but a functional regulator of metastatic progression. It may support local invasion, immune escape, vascular access and intravasation, and maintenance of plastic or stem-like states that facilitate dissemination and early metastatic seeding.

First, the TME may facilitate local invasion and release from the primary tumor. Although the role of cancer-associated fibroblasts has historically been questioned in mesenchymal tumors [33], non-malignant stromal populations are present in sarcomas [34] and may influence extracellular matrix organization, local tissue remodeling, and tumor cell migration. In bone sarcomas, the hypoxic bone microenvironment can induce the HIF-1α/CXCR4 axis, which has been implicated in tumor progression and metastasis, notably in osteosarcoma [35]. Even modest reductions in oxygen tension can promote dissemination by stimulating tumor cell migration and invasiveness [36]. In addition, osteoclast-mediated bone remodeling during osteosarcoma development can release growth factors such as FGF, TGF-β, and IGF1, thereby generating a local milieu that may support invasion, survival, and subsequent dissemination [37].

Second, the TME may promote immune evasion during early metastatic progression. The sarcoma TME appears highly immunosuppressive because of abundant macrophages, specifically M2-like tumor-associated macrophages. High neutrophil density and low T-cell levels also contribute to this immune-hostile TME [38]. Unlike epithelial cancers, many mesenchymal malignancies have low tumor mutational burden, which contributes to poor immunogenicity. Together, these features may promote tumor growth and metastatic progression while limiting current immunotherapeutic efficacy in many sarcoma subtypes.

Third, the TME may facilitate vascular access and intravasation. Local angiogenesis, endothelial activation, perivascular remodeling, and hypoxia-driven signaling may create permissive interfaces through which sarcoma cells enter the circulation. Route selection is discussed further in Section 5.

Finally, the TME may help maintain plastic or stem-like states that support metastatic competence. MSCs within the TME may support osteosarcoma CSCs, promoting tumor progression and metastasis [39]. However, it is unclear whether this effect is sarcoma-specific or also occurs in carcinoma CSCs. Therefore, the contribution of the TME to sarcoma dissemination remains to be fully elucidated.

## Migration and invasion in mesenchymal tumors

A malignant cell must acquire migratory and invasive properties to escape its stroma and, ultimately, metastasize. In carcinomas, both properties are tightly linked to EMT, a reversible transition between the epithelial and mesenchymal phenotypes. This cellular reprogramming involves partial to complete loss of epithelial features and partial to complete gain of mesenchymal characteristics. Epithelial cells undergoing EMT have reduced intercellular adhesion due to the loss of E-cadherin and other cell–cell interaction molecule expression, loss of polarity and epithelial cytoskeletal intermediate filament expression, and reduced proliferation. In contrast, they gain expression of mesenchymal adhesion molecules, including N-Cadherin, the mesenchymal intermediate filament vimentin, and cytoskeletal reconfiguration that favors migration and invasion. EMT and its reverse process, the mesenchymal-to-epithelial transition (MET), play crucial physiologic roles in embryogenesis, as well as in metastatic dissemination and drug resistance of epithelial tumors. Because sarcoma cells originate from mesenchymal tissue, they do not possess the epithelial differentiation features of carcinomas. Hence, it was believed that sarcoma cells do not disseminate via EMT-MET. However, some investigators now hypothesize that, due to their plasticity, sarcoma cells can undergo a mild form of EMT-MET [40]. Sannino et al. proposed that the clinical aggressiveness of sarcomas is partially due to their intermediate phenotype between mesenchymal and epithelial states, which allows them to undergo partial MET or EMT under specific conditions [41]. This notion is supported by the dynamic expression of the epithelial cellular adhesion molecule (EpCAM) protein during pediatric sarcoma evolution and its link to patient outcomes [42]. In Ewing sarcoma, the migratory propensity of the tumor cells is influenced by their distinct states, which depend on fluctuations in expression of EWSR1-FLI1, an aberrant transcription factor that drives oncogenic transformation of permissive cells. These fluctuations may be sufficient to influence cell dissemination by regulating the degree of the mesenchymal phenotype. High EWSR1-FLI1 expression is associated with tumor cell proliferation, whereas low EWSR1-FLI1 expression appears to induce migratory and invasive behavior. Cells with low EWSR1-FLI1 levels thus appear to more loosely mimic the phenotype of the MSC cell of origin and may be more efficient in forming metastasis [43]. Bierbaumer et al. demonstrated that, in cells with low EWSR1-FLI1, the TAZ-YAP pathway, a master coordinator of EMT, is activated and induces cytoskeletal changes in the cells that correlate with their increased metastatic proclivity [44]. Current evidence suggests that sarcomas can exhibit variable and partial EMT-MET, which may contribute to their survival within the circulation, colonization of secondary sites, and ultimately, development of metastasis [45].

EMT induction involves tumor cell–autonomous signals and is influenced by heterotopic signals from the stroma [46]. Any source of tissue remodeling (e.g., hypoxia, inflammation, repair) results in the extracellular matrix releasing a plethora of cytokines that contribute to EMT. Thus, chemotherapy, which may be insufficient to destroy all the tumor cells, may induce EMT by causing necrosis and inflammation in surviving cells and contribute to chemotherapy-induced metastasis.

## Intravasation and route of metastatic spread

Previously, it was assumed that sarcomas spread via blood vessels but carcinoma cells disseminate more readily through the lymphatics. Because the lymphatics and the blood vasculature are interconnected through the thoracic duct and the right lymphatic duct [47], the TNM cancer staging system assumes that primary tumor cells (T) spread first to regional lymph nodes (N) before giving rise to distant metastases (M) [48]. In carcinomas, lymph node metastases are critical for tumor staging and prognosis. However, this notion has been challenged because distant metastasis can occur despite the absence of lymph node metastasis, and several clinical trials have shown that surgical resection of positive lymph nodes does not always improve patient outcome [49]. These observations raise questions regarding earlier assumptions as to the dynamics of tumor dissemination and suggest that positive lymph nodes may not invariably serve as a source of metastasis. Indeed, mathematical models indicate that the size and timing of metastases in distant organs are inconsistent with seeding from lymph node metastases, which would require additional time to first establish and expand, and instead support direct dissemination from the primary tumor [50]. Positive lymph nodes may represent a terminus for disseminated tumor cells in some malignancies, but they most likely signal a concomitant spread from the primary tumor through the bloodstream.

**Fig. 2:**
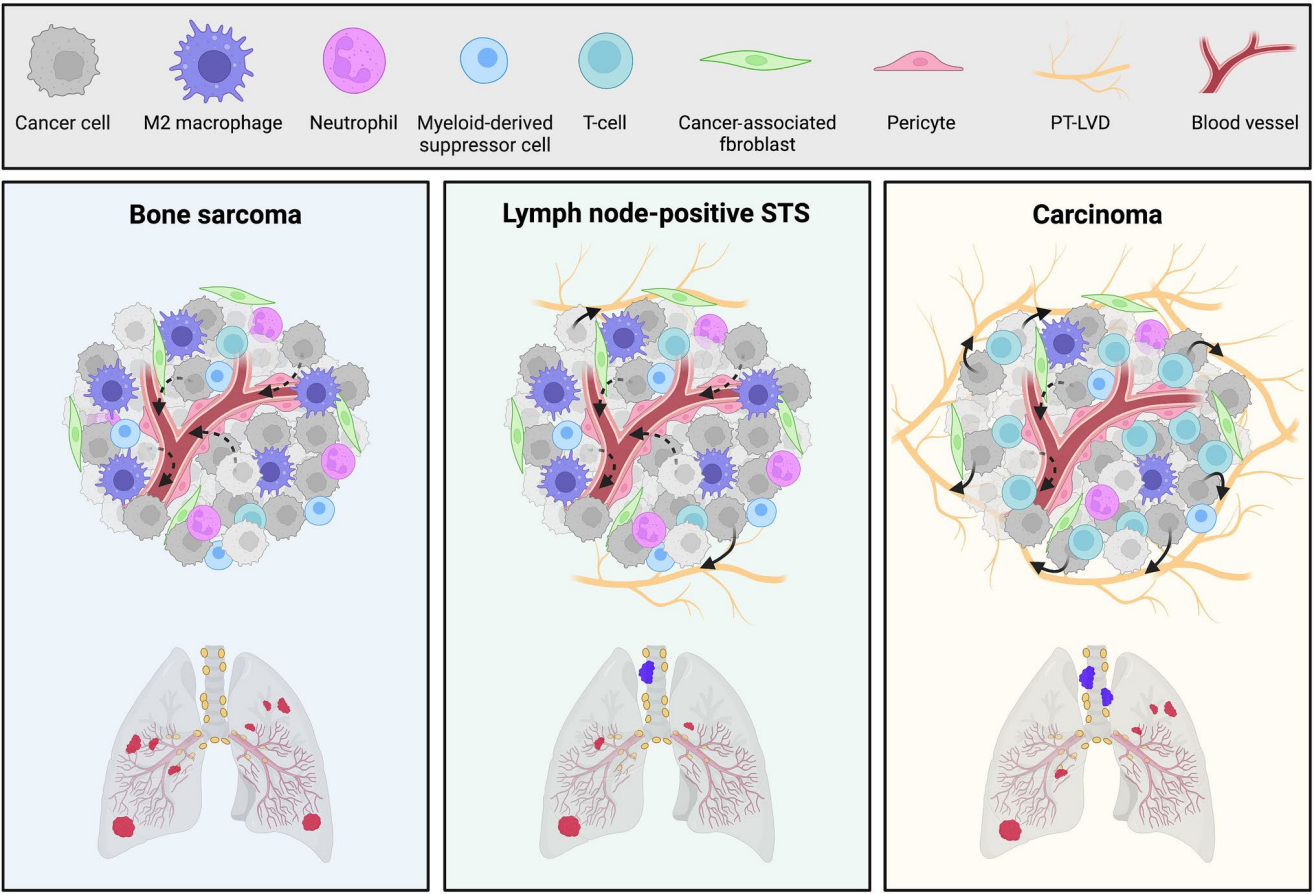
While sarcomas were thought to spread through blood vessels and carcinomas through lymphatics, accumulating evidence suggests that the choice of dissemination route may be more complex. Accessibility to blood versus lymphatic vasculature, differences in peritumoral lymphatic vessel density, and intrinsic tumor cell features (e.g., mesenchymal versus epithelioid traits) may all influence whether a tumor cell enters the bloodstream or the lymphatic system. Blood vessels impose a stricter barrier to intravasation than lymphatics, but sarcoma cells are believed to be naturally equipped for hematogenous spread, whereas carcinoma cells more readily exploit the lymphatic route. Exceptions exist, as certain epithelioid-like soft tissue sarcoma subtypes can metastasize to lymph nodes. This figure was created with BioRender

It is largely unclear why carcinomas tend to develop lymph node metastases more frequently than sarcomas. However, a few sarcoma histotypes, including epithelioid sarcoma, clear cell sarcoma, angiosarcoma, and rhabdomyosarcoma, do have a proclivity to disseminate via lymphatic vessels [51, 52]. Interestingly, these tumors, or their more differentiated subtypes, bear an epithelioid phenotype [53] (Fig. 2). For instance, about 30% to 40% of rhabdomyosarcomas express epithelial markers [54].

### Accessibility to lymphatic versus blood vasculature

Most sarcomas and carcinomas can drive angiogenesis, which facilitates dissemination via the bloodstream, but not lymphangiogenesis. Although lymphatic and vascular vessels share the same embryonic origin and respond to several common growth factors, it is unclear why the dynamics and amplitude of angiogenesis and lymphangiogenesis are not commensurate during tumorigenesis in most cancer types. Moreover, in contrast to the blood vasculature, lymphatic vessels are not mandatory for tumor cells to survive and proliferate [55], and lymphangiogenesis or intratumoral lymphatic vessels are not absolutely necessary for carcinoma cells to invade local lymph nodes [56]. This suggests that only peritumoral lymphatics are required for lymphatic dissemination [57]. Lahat et al. showed that lymph node–positive soft-tissue sarcomas (STS) displayed higher peritumoral lymphatic vessel density than lymph node–negative counterparts [58]. Moreover, peritumoral lymphatic vessel density in STS is lower compared to breast carcinoma, which partly explains the paucity of STS lymph node metastasis [58]. In STS, high expression of vascular endothelial growth factor (VEGF)-D or forced overexpression of VEGF-C, two major regulators of lymphangiogenesis, was correlated with the propensity for lymph node metastasis [57, 59]. This highlights the importance of lymph vessel density and accessibility for metastatic dissemination. In bone sarcomas, the paucity of lymphatic channels in the bones could explain the scarcity of positive lymph node disease. Finally, sarcomas are highly angiogenic and vascular tumors [60]. Ample blood accessibility may play a role in its preferred hematogenous spread route, although results are conflicting regarding the use of microvessel density as a prognostic factor in mesenchymal malignancies [61, 62].

### Intravasation mechanisms

Both passive and active modes of intravasation have been described. In the passive mode, cancer cells penetrate the vasculature as a result of the tumor mass disrupting blood vessels. In contrast, active intravasation implies detachment and migration of single or clusters of tumor cells toward blood vessels, often in response to chemotactic cues [63]. Gene expression studies have highlighted differences between lymphatic and blood endothelium, notably in their expression of chemotactic factors, which could partially explain why a tumor cell selects one route instead of the other [64, 65]. However, consistent with observations in carcinomas, EMT-MET has been proposed to influence the metastatic route in sarcomas [41]. In the rare cases where osteosarcoma metastasizes to lymph nodes [66], the tumor cells display an epithelioid phenotype [67]. The pericytes, smooth muscle cells, and a well-defined basement membrane envelop blood vessels and constitute a robust barrier to invasion and penetration by exogenous cells. Thus, single carcinoma cells that successfully penetrate the blood vasculature exhibit robust EMT features, including a dynamic cytoskeleton that ensures migratory properties, and expression of adhesion receptors and proteolytic enzymes that can be concentrated at points of contact with the vessel wall. Similarly, groups of carcinoma cells that penetrate blood vessels are led by one or several cells with EMT features. In contrast, lymph vessels do not have a strong barrier and are easier to penetrate than blood vessels because they lack the junctions characteristic of vascular endothelial cells, are not surrounded by a sheath of pericytes or smooth muscle cells, and have only a poorly defined basement membrane [47]. Tumor cells therefore do not require the same spectrum of mesenchymal features to penetrate the lymphatics as they would need to penetrate blood vessels. Moreover, the slow circulation of lymph does not subject tumor cells to shear stress [64], providing a milieu in which cells that may be unfit for survival in blood vessels may readily survive on their journey to lymph nodes. Having intrinsic mesenchymal features, most sarcoma cells may be equipped to penetrate blood vessels — perhaps successful carcinoma cells mimic what sarcoma cells do naturally. Lymphatic dissemination may be the route of less well-equipped tumor cells, which may reflect the tendency of carcinoma cells to be more successful in initiating lymph node metastases than in forming blood-borne metastases.

## CTCs

CTCs, which are malignant cells detected in peripheral blood, are distinct from disseminated tumor cells (DTCs) that are already located in secondary organs and are derived from a subpopulation of CTCs [68]. In carcinomas, the recognition and investigation of CTCs have resulted in tools that facilitate earlier detection of a tumor, better determination of prognosis and therapeutic response, as well as a more accurate prediction of overall and progression-free survival [69]. Less CTC research has been carried out in sarcomas, partly because fresh blood samples are limited and sarcoma CTC–specific markers remain difficult to define. Unlike carcinoma CTCs, sarcoma CTCs often lack epithelial differentiation and share mesenchymal features with normal stromal or circulating mesenchymal cells. Thus, the key challenge is not only detecting rare cells but also validating markers that are sufficiently specific while preserving the phenotypic diversity of sarcoma CTCs. However, sarcoma CTCs in mesenchymal tumors could help study metastasis and monitor response or recurrence [7, 70].

### Isolating sarcoma CTCs

Because CTCs are rare in peripheral blood (approximately 1 CTC per 10^6^–10^9^ blood cells [71], equal to only a few cells per milliliter of blood) and have a heterogeneous and plastic phenotype, isolating CTCs is a challenging task. Moreover, most approaches used to isolate carcinoma CTCs rely on epithelial antigen-specific antibodies and cannot be used in sarcomas. Methods to isolate sarcoma CTCs are based on criteria that include cell size [72], expression of common mesenchymal markers, such as vimentin [73], sarcoma subtype-specific markers, including signature chromosomal translocations [74], and osteoblast-related genes for bone tumors [75]. However, these methods have limitations. Excluding cells based on size alone is limited because it fails to include any specific phenotypic markers. Vimentin expression is not specific for sarcomas; it is also expressed in normal mesenchymal cells, including circulating MSCs and carcinoma CTCs that have undergone EMT [76].

The PCR-based methodology to detect chromosomal translocations can only be applied to a handful of rare sarcoma subtypes, and its sensitivity depends on the expression level of the corresponding transcript within the cells. This technique does not seem suitable for Ewing sarcoma cells, where EWSR1-FLI1 expression fluctuates from barely detectable to high levels and was recently proposed to be inversely correlated to metastatic proclivity [43]. Nevertheless, this is the most used approach in Ewing sarcoma. Flow cytometry techniques are mainly limited by the lack of histology-specific markers and the difficulty of detecting CTC clusters based on the gating on single cells. Thus, meaningful progress will require multimodal identification strategies rather than single-marker capture, combining physical enrichment with subtype-specific genetic alterations, copy-number profiles, fusion transcripts when applicable, and validated surface marker panels.

### Characterizing sarcoma CTCs

In carcinomas, characterizing CTCs has highlighted their plasticity and phenotypic heterogeneity [77], which reflects the mutational and epigenetic modification spectra within the primary tumor and its metastases. CTC heterogeneity has been linked to prognosis and treatment response, with higher heterogeneity associated with poorer prognosis, increased metastatic potential, and resistance to therapy [78], perhaps because they mirror and provide a snapshot of how the corresponding tumor evolves and progresses. In contrast, almost nothing is known about sarcoma CTC properties. The presence of stemness characteristics in sarcoma CTCs remains to be established, as does the extent of their potential as MICs.

CTC clusters in carcinomas are potent initiators of metastases and are linked to adverse prognosis [79]. Recently, the enhanced metastatic potential of CTC clusters in breast cancer has been proposed to be linked to the hypomethylation of binding sites for stemness- and proliferation-associated transcription factors, including OCT4, SOX2, NANOG, and SIN3A [80]. Although CTC clusters have been described in sarcoma patients, their prognostic significance, compared to that of single CTCs, remains unknown largely due to the small number of samples examined.

Beyond enumeration, future studies should functionally characterize sarcoma CTCs and CTC clusters to determine whether specific subsets display stemness, plasticity, anoikis resistance, immune-evasive features, or true metastasis-initiating capacity.

### Sarcoma CTC survival

In carcinomas, most CTCs perish due to the numerous obstacles they encounter during their journey, including hydrodynamic shear forces, recognition by the immune system, and the loss of attachment to substrate. In normal epithelial cells and a variable fraction of carcinoma cells, these obstacles trigger anoikis. Resistance to anoikis, a mode of apoptotic cell death regulated by integrins [81], has been proposed to constitute a critical step in the survival of sarcoma CTCs and, by extension, in sarcoma metastasis. Carcinomas and sarcomas share common signaling pathways involved in anoikis resistance, including activating PI3K/Akt and Ras/ERK survival pathways [81, 82]. However, cells may use various ways to trigger such survival signals may be sarcoma- or sarcoma subtype–specific [83, 84]. Recently, Zhang et al. demonstrated in Ewing sarcoma that the signature EWSR1-FLI1 fusion oncoprotein directly upregulates the cell surface protein IL-1 receptor accessory protein (IL1RAP) to inhibit anoikis by controlling reactive oxygen species detoxification. IL1RAP inactivation in an *in vivo* model correlated with a marked decrease of lung metastases [85]. This decrease, along with the minimal expression of IL1RAP in pediatric and adult normal tissues, suggests that IL1RAP may be a candidate therapeutic target in Ewing sarcoma.

In the bloodstream, sarcoma CTCs must resist the immune system, and in particular, natural killer cells [86]. Similar to carcinoma cells, circulating sarcoma cells can interact with and become shielded by platelets, which protect them from immune cell aggression and shear stress, augmenting their likelihood to form metastatic colonies [87].

### Clinical significance of sarcoma CTCs

In sarcoma animal models, the number of CTCs correlates with the evolution of the disease — CTCs increase while the primary tumor is growing and then rapidly decline once the tumor is removed. Mice that experience metastatic relapse have an increase in CTCs [88]. Clinical studies seem to corroborate these *in vivo* observations: CTCs in the bloodstream of sarcoma patients seem to indicate poor prognosis [75, 89]. Hayashi et al. found that CTCs in patients with no radiologic evidence of disease was correlated with impending relapse, suggesting that changes in CTC numbers may serve as an indicator of treatment response and relapse [88]. In a recent pilot study, CTCs bearing aneuploidy were identified as a prognostic factor for worse progression-free survival and overall survival in STS [90].

Although most of these observations align with those from carcinoma CTC studies [91], they are based on a limited number of small clinical studies. More robust trials are currently underway to validate them.

### Other promising biomarkers

Blood-based biomarkers collected by minimally invasive liquid biopsy, such as circulating tumor DNA (ctDNA), microRNAs, and exosomes, complement the use of CTCs for monitoring, predicting, and improving our understanding of metastasis. ctDNA is typically shed from dying primary, circulating, or metastatic cancer cells [92]. Shulman et al. showed that detectable ctDNA in newly diagnosed Ewing sarcoma patients is associated with metastatic disease and poor outcome [93]. Furthermore, a rapid decrease in ctDNA bearing the fusion gene *EWSR1-FLI1* has been correlated with induction chemotherapy, whereas ctDNA increase heralded tumor relapse [94]. Consequently, ctDNA could be a valuable marker for assessing treatment response in sarcomas. Identification and quantification of certain microRNAs have been related to the diagnosis, grading, staging, and clinical outcomes of several sarcoma subtypes [95]. However, their widespread applicability in the clinical setting remains to be demonstrated. Exosomes deliver various proteins and polynucleotides to target cells by fusing with their lipid bilayer, and they have been linked to TME modulation, tumor progression, and metastasis [96].

Liquid biopsy could enable longitudinal tracking of tumor burden, treatment response, relapse, and cancer-related genetic or epigenetic changes in sarcoma. Although no liquid biopsy approach is yet broadly implemented in routine sarcoma care [97], clinical translation efforts are underway. In particular, the FOSTER (Fight Osteosarcoma Through European Research) and Euro Ewing Consortium statement proposed harmonized recommendations for the collection, processing, storage, and analysis of biological samples, including blood-based liquid biopsy material, in osteosarcoma and Ewing sarcoma [98]. Such harmonization will be essential to move liquid biopsy from proof-of-concept studies toward reproducible monitoring and biologically informative clinical trials.

## Extravasation

After a variable transit time in the circulation, which may last only seconds to minutes [99], CTCs leave the vasculature by extravasation in order to settle within secondary organs.

### CTC immobilization

Several parameters, including anatomical, molecular, and mechanical features, are believed to contribute to determining where a CTC becomes immobilized. *In vivo* video microscopy has shown that most CTCs arrest by size restriction in the first capillary bed that they encounter [100]; the diameter of capillaries (3–8 µm) is smaller than that of many cancer cells (10 µm or more) [101]. However, metastatic spread cannot be explained by vascular anatomy alone, as CTCs can become immobilized in vessels larger than capillaries [102]. These CTCs actively interact with vessel walls via their adhesion receptors, including integrins and selectins, which recognize their respective ligands on the surface of vascular endothelial cells and in the underlying basement membrane [103]. Sarcoma and carcinoma CTCs may be prone to arrest at different sites along the endothelium because the adhesion receptor repertoires of sarcoma and carcinoma CTCs may be distinct and because endothelial cells in different organs express diverse sets of adhesion receptor ligands. However, possible differences between sarcoma and carcinoma CTC tropism for endothelia of different organs must be confirmed by large scale analysis of sarcoma CTC phenotypes, which is currently lacking.

Hemodynamic forces also play an important role in cell adhesion, endothelium remodeling, and extravasation of cancer cells. CTCs have been shown to require a slow flow rate of 400–600 µm/s for successful extravasation. In patients, a larger number of brain metastases are detected in regions with low cerebral blood perfusion levels [104]. Whether hemodynamic forces affect sarcoma and carcinoma CTCs in the same way remains to be determined.

### Extravasation mechanisms

**Fig. 3:**
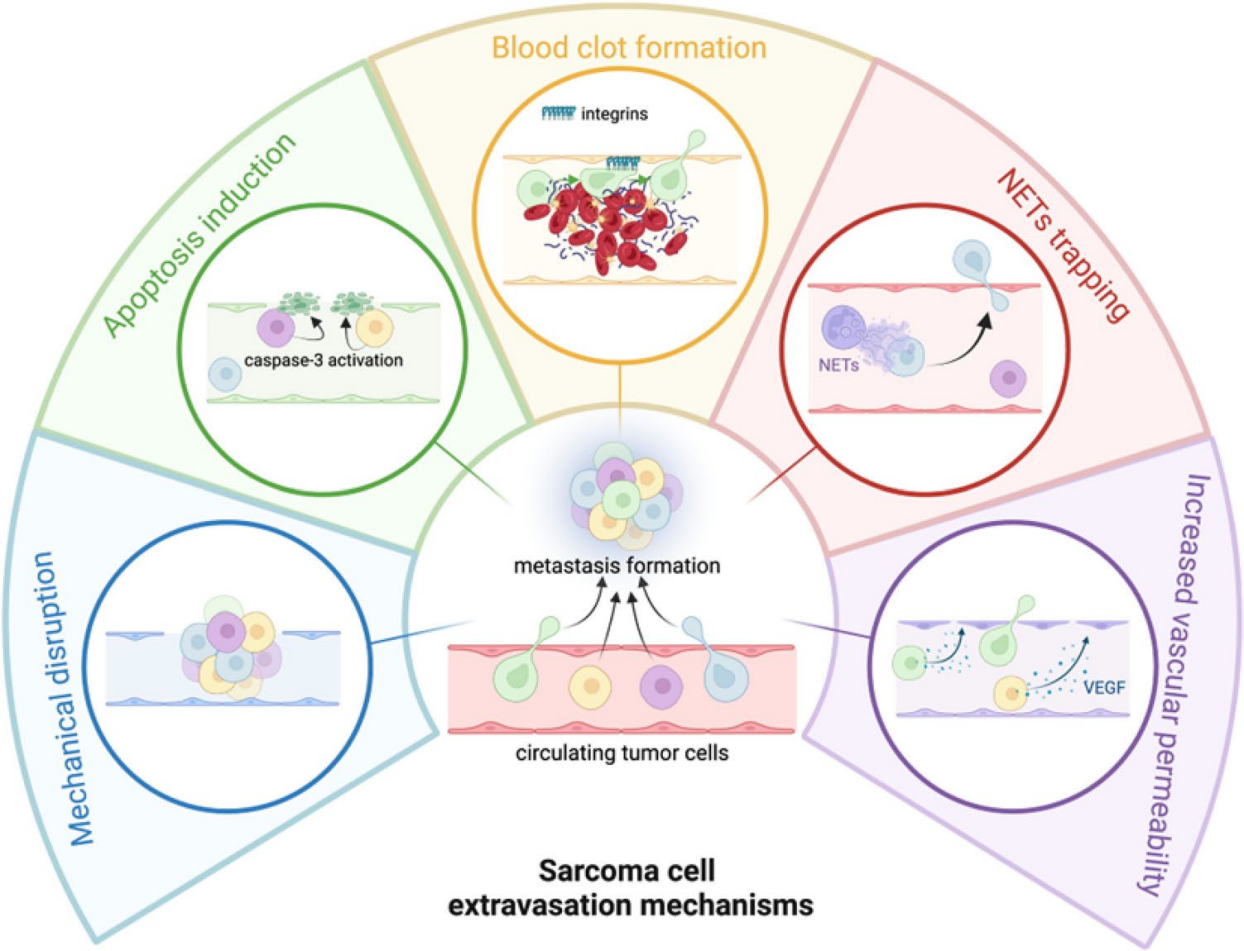
Sarcoma cells can extravasate through multiple non-mutually exclusive mechanisms. These include mechanical disruption of the endothelial barrier; apoptosis induction in endothelial cells; secretion of VEGF leading to increased vascular permeability; recruitment of platelets and blood clot formation; and neutrophil extracellular traps (NETs) trapping. These distinct mechanisms converge to promote metastasis formation, with the route of extravasation influenced by both the intrinsic biological properties of CTCs and the permissiveness of the local endothelium. This figure was created with BioRender

Various mechanisms facilitate tumor cell extravasation, ranging from mechanical disruption of capillary walls to processes mimicking the extravasation of leukocytes with or without the help of white blood cells and platelets (Fig. 3). A fibrosarcoma model showed that endothelial integrity is disrupted by intravascular proliferation of tumor cells that outgrow the vessels they were in or induce apoptosis of endothelial cells [102]. The high level of VEGF secreted by osteosarcoma tumor cells has been correlated with increased vascular permeability and extravasation [105]. Most studies conducted on sarcoma cell extravasation have suggested a link between the expression of certain genes (e.g., *MGP*, *CYGB*, *ICAM1*) and extravasation of malignant cells, but these descriptive studies did not establish a cause-and-effect relationship or provide a mechanistic explanation [106].

Platelet recruitment and blood clot formation may promote sarcoma cell extravasation by activating integrins and forming invadopodia, which facilitate cell penetration into narrow inter-endothelial spaces [107] and endothelial adhesion [108]. Tumor-associated macrophages may help some sarcoma cell types cross endothelial barriers, but the exact mechanism remains elusive [109]. Neutrophil extracellular traps (NETs) have also been proposed to facilitate CTC extravasation. NETs are web-like structures made of chromatin and neutrophil proteins released by activated neutrophils [110]. They were initially observed to trap pathogens during immune responses to infection, but their role in cancer immunoediting and metastasis has been recently highlighted [111]. NETs can be deployed to capture CTCs that express diverse repertoires of integrins, thus promoting metastatic extravasation [112]. In Ewing sarcoma, NET formation by tumor-associated neutrophils was associated with early relapse and metastatic disease [111].

Tumor cells can exit the circulation by several mechanisms, and the mechanism used by any given CTC depends on a combination of its own biological properties [100] and those of the local endothelium. Bone marrow and liver capillaries, which have a fenestrated endothelium with a discontinuous basal lamina, are more permissive exit sites than pulmonary capillaries, which are lined by endothelial cells held together by tight junctions and a continuous basement membrane [113]. Based on their inherent mesenchymal features, most sarcoma cells could extravasate wherever they become immobilized. However, there are too few studies addressing sarcoma cell extravasation to draw definitive conclusions.

## Colonization: the bottleneck of metastasis

The location at which a cancer cell extravasates is determined in part by the anatomy of the blood circulation and the composition of the vascular wall. Although many tumor cells may reach distant organs, only a minority survive, adapt to the foreign microenvironment, and expand into clinically detectable metastases. In sarcoma, this bottleneck is particularly relevant because metastatic dissemination is strongly biased toward the lung. Up to 20% of patients diagnosed with soft-tissue sarcoma and approximately 40% of patients with bone sarcoma present with isolated pulmonary metastatic disease [62, 114]. As a result, sarcoma metastasis studies have often focused solely on the lung.

However, lung tropism remains insufficiently explained. A purely anatomical model, in which circulating sarcoma cells are mechanically trapped in the first capillary bed they encounter, is unlikely to account for the full biology of sarcoma metastasis. Indeed, lung vasculature is less permissive for extravasation than that of organs such as the liver or bone marrow, suggesting that successful pulmonary metastasis requires additional tumor–host interactions. Thus, sarcoma lung colonization should be viewed not only as a consequence of vascular filtering, but also as the result of organ-specific survival cues, stromal support, and intrinsic adaptations that allow disseminated tumor cells to overcome early attrition.

Several extrinsic mechanisms may contribute to organ-specific colonization. Landuzzi et al. found that Ewing sarcoma metastatic sites, including lung, bone, and bone marrow, are stem cell factor–rich microenvironments. Ewing sarcoma cells express the stem cell factor receptor c-kit, so stem cell factors were suggested to be a strong organ-specific chemoattractant for Ewing sarcoma [115]. Consistent with this observation, chemokines and their receptors have been more broadly implicated in organ-specific dissemination of sarcoma cells [116]. Tissue-specific survival and proliferation advantages of metastatic cells are strengthened by other mechanisms, such as interactions with resident lung cells and cells recruited to the metastatic niche.

Colonization also depends on cell-autonomous adaptations. Several studies have identified genes or gene signatures involved in the colonization of a specific organ, such as lung or brain (128, 129). Using a genomic approach to reveal metastasis-associated genes, Khanna et al. identified ezrin, which links the cell membrane to the actin cytoskeleton, allowing the tumor cell to interact with its microenvironment [117]. This process appears to be necessary for osteosarcoma lung metastatic colonization. Ezrin phosphorylation prevents apoptosis of DTCs by activating protein kinase C, which allows their survival during the bottleneck period of early attrition after the delivery of tumor cells to the lung [117]. In other sarcomas, ezrin has also been associated with metastasis and poor outcomes, suggesting a potential therapeutic target [118]. However, very little concordance has been found among the different organ-specific metastasis gene signatures in particular sarcoma subtypes or in sarcomas in general. One partial exception is the secretory apolipoprotein J/clusterin (sCLU), which was highlighted in two different lung-specific metastatic signatures in osteosarcoma [119, 120]. sCLU overexpression in osteosarcoma has been significantly correlated with lung metastasis in patients [121]. Overall, these observations suggest that lung colonization may not depend on a universal sarcoma-specific metastatic program, but rather on different combinations of tumor-intrinsic traits and microenvironmental interactions.

Epigenomic modifications also help sarcoma cells colonize distant organs. Morrow et al. showed in osteosarcoma that gene expression recapitulating the metastatic phenotype is regulated by a shift in the enhancer epigenome. Altered enhancer activity induces the expression of several genes believed to be required to overcome the barriers to metastatic colonization of secondary organs [122]. This supports the notion that primary cancer cells may already possess the genetic equipment required to metastasize.

### Adapting to a new microenvironment

After extravasation, disseminated tumor cells must adapt to a hostile foreign tissue. They encounter changes in oxygen tension, nutrient availability, pH, oxidative stress, and immune pressure. The endoplasmic reticulum senses several forms of cellular stress and can initiate either adaptive survival programs or cell death [123]. Highly metastatic cells can adapt their endoplasmic reticulum stress response by upregulating chaperone proteins to resist cell death [124]. Recently, Zhang et al. described another cell-autonomous reprogramming that favors osteosarcoma lung metastases, termed mesenchymal-to-fibrosis transition [125]. In this model, host-derived fibroblast growth factor activates an FGFR2–fibronectin axis in osteosarcoma stem cells, inducing the production of fibrillar matrices independently of tissue-resident fibroblasts. This self-generated fibrotic matrix supports survival and proliferation of osteosarcoma stem cells. Interestingly, this phenotype was not observed in epithelial tumor stem cells under similar experimental conditions, suggesting that fibrogenic reprogramming may be particularly relevant to mesenchymal cancers [125].

**Fig. 4:**
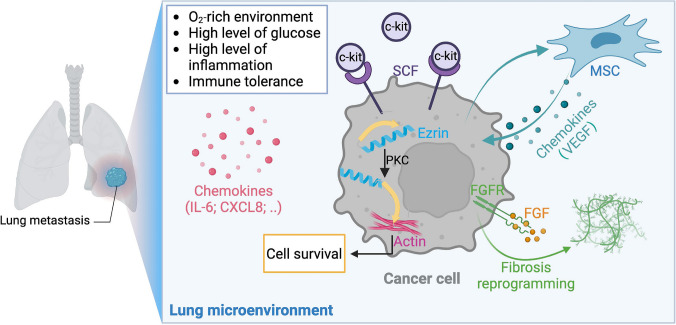
Following extravasation, disseminated sarcoma cells face major obstacles to survival and growth in secondary organs. Sarcomas predominantly metastasize to the lungs. Both extrinsic cues, such as chemokine- and cytokine-rich microenvironments or support from resident stromal cells, and intrinsic, cell-autonomous adaptations (e.g. ezrin-PKC pathway) have been implicated in overcoming early attrition. This figure was created with BioRender

Sarcoma predilection for lung colonization suggests favorable tumor-host interactions that support metastatic cell survival and proliferation. Gross et al. underscored this notion by identifying interleukin-6 and interleukin-8, a bidirectional cytokine signaling axis between metastatic osteosarcoma and lung parenchyma cells that promotes metastatic progression [116]. Moreover, osteosarcoma cells can manipulate resident lung tissue MSCs by secreting extracellular vesicles to facilitate metastasis formation [126]. MSCs were shown to help osteosarcoma cells settle in and colonize the pulmonary microenvironment by secreting chemokines and VEGF [127]. In contrast, in Ewing sarcoma and Kaposi sarcoma, MSCs display an anti-tumorigenic effect [128, 129]. These seemingly opposing properties may be attributed to the heterogeneity of MSC phenotypes according to which tissue they are derived from (e.g., lung versus bone marrow), a specificity of the tumor type, or differences in experimental design (Fig. 4).

In carcinoma, DTCs are believed to be located in an environment favorable to stem cell maintenance, often referred to as a niche, which supports their self-renewal necessary to initiate metastasis. Such niches are mostly perivascular, which facilitates the supply of oxygen, nutrients, and paracrine factors from the activated endothelium [130]. Whether equivalent niches exist in sarcoma remains largely unknown. Similarly, although MET signaling has been proposed as a pathway that reinitiates proliferation during metastatic colonization in carcinomas [131], its role in sarcoma metastasis remains unclear, despite evidence that MET activity may be regulated by master transcriptional programs shared with carcinoma metastasis [132].

### Escaping the immune defenses of infiltrated tissue

DTCs are vulnerable to the immune system, and most of them succumb to cytotoxic T-cell or natural killer cell action. In carcinomas, disseminated cancer cells use several well-established mechanisms to avoid immune detection or inhibit anti-tumor immune activity [133]. Whether sarcoma cells elicit similar immune responses and use the same or distinct mechanisms to evade them remains to be determined.

### Organ-specific metastatic traits

Some organs, such as the liver, lung, brain, and bone, are more prone to hosting metastases, whereas secondary tumors almost never grow in skeletal muscle, despite its abundant blood supply. The requirements for metastatic cells to colonize secondary sites may vary from organ to organ.

Bone metastases deserve specific consideration in sarcoma because it is both a frequent primary site for bone sarcomas and a clinically relevant metastatic site, including in some adult STS. Although pulmonary metastases remain the dominant pattern of hematogenous spread in sarcoma, bone illustrates that metastatic tropism cannot be explained by vascular filtering alone. The bone represents a specialized metastatic microenvironment, characterized by sinusoidal vessels, active extracellular matrix remodeling, osteoblast–osteoclast coupling, mesenchymal stromal compartments, and abundant chemokine and growth-factor signals. These features provide both docking cues and survival signals for disseminated tumor cells, consistent with the “seed and soil” model of organ-specific metastasis [134]. This may be particularly relevant in pediatric and mesenchymal sarcomas, in which malignant cells share developmental and stromal programs with the bone microenvironment. The CXCL12/CXCR4 axis provides one example of such organ-associated compatibility. CXCL12-rich organs, including bone marrow, may attract or retain CXCR4-positive sarcoma cells, while CXCR4 signaling can promote chemotaxis, endothelial adhesion, matrix metalloproteinase expression, and metastatic competence [134]. In osteosarcoma and other pediatric sarcomas, hypoxia-driven CXCR4 expression, VEGF signaling, matrix metalloproteinase activity, and interactions with bone marrow-derived mesenchymal stromal cells further support a model in which disseminated sarcoma cells are not passively trapped in bone, but are selected and supported by a permissive mesenchymal microenvironment [135]. Thus, bone metastasis in sarcoma may be viewed as an organ-conditioned metastatic phenotype, in which tumor-intrinsic mesenchymal plasticity converges with the trophic, stromal, and remodeling properties of the bone marrow microenvironment.

This principle is not restricted to bone. Other organs impose distinct selective pressures that metastatic cells must overcome in order to survive and expand. To initiate metastasis in brain tissue, for instance, lung and breast cancer cells must produce serpins to counteract the cytotoxic activity of astrocytes [136]. Tumor cells that frequently spread to the lungs, such as sarcoma, renal cell carcinoma, colorectal cancer, melanoma, and breast carcinoma cells, may share molecular traits that are relevant to colonization of and growth within lung tissue. For example, tenascin C, an extracellular matrix glycoprotein, has been shown to be important for lung engraftment of breast tumor cells [137]. It has also been implicated in pulmonary metastases of melanoma, Ewing sarcoma, osteosarcoma, and primitive neuroectodermal tumor [138]. In renal cell carcinoma and colorectal cancer, tenascin C has been linked to metastasis, though not specifically to the lungs [139]. However, organ-specific molecular signatures for metastases have not been identified in sarcomas, suggesting that different combinations of cancer cell features may be needed for metastasis to different organs. Tang et al., while tracing sarcoma evolution, observed that a single metastatic clone can generate metastases in diverse organs [19]. These data suggest that a metastatic sarcoma cell may harbor features that allow survival and growth in various microenvironments. However, it remains to be determined whether these observations are generally attributable to sarcomas or limited to the model used in the study.

### Dormancy

Once the secondary organ is colonized, DTCs may become dormant, remaining quiescent for weeks to years before proliferating again and inducing macroscopic metastasis. The length of this latency period depends on numerous factors, which may include the tumor type, cell-intrinsic pathways, and signaling from the host microenvironment. Late metastatic relapse, generally defined as detectable metastasis after a disease-free interval of more than 5 years, is uncommon in sarcomas [140], except in synovial sarcoma [141]. Synovial sarcomas grow slowly, which could partly explain the relatively high incidence of late metastases independent of dormancy [141]. In slow-growing cancers, dormancy is less likely if one assumes that growth rates of metastases are similar to those of primary malignancies [142]. On the other hand, dormancy may likely explain late metastases that occur in fast-growing tumors.

Cancer dormancy remains poorly understood. Mechanisms suggested to be implicated in sarcoma cell metastatic latency include angiogenic dormancy, which requires an angiogenic switch to induce cellular re-awakening [143], p38 MAPK pathway activation [144], and immunosurveillance mediated by T lymphocytes and natural killer cells to restrain spontaneous metastasis [145]. However, current understanding of sarcoma cell dormancy is limited, and it remains unclear to what extent sarcomas are subject to this phenomenon.

## Treating sarcoma metastasis

As is the case for carcinomas, there is no broadly effective sarcoma metastasis–curbing treatment, and disseminated disease causes most sarcoma-related deaths. The three main systemic approaches used to treat metastases are chemotherapy, targeted therapy, and immunotherapy. Although the last two strategies improved survival in disseminated melanoma or metastatic lung and breast carcinomas [146], they have not decreased metastatic sarcoma mortality, highlighting the need for new approaches. Chemotherapy is the mainstay and often the sole option for metastatic sarcoma, but it remains largely inefficient and has substantial side effects [147]. Furthermore, evidence suggests that some chemotherapies may trigger metastasis by increasing intravasation and generating genetic alterations that favor resistant clones [148]. In certain cases, surgery can be curative for patients with metachronous pulmonary metastases, if all lesions are resectable [149]. Radiotherapy and interventional radiological ablation methods are used to locally control sarcoma oligometastatic disease and improve overall survival.

The biological principles discussed in this review suggest that future treatment strategies should target metastatic competence rather than tumor burden alone. If sarcoma metastasis depends on plastic, metastasis-initiating states, then therapeutic approaches aimed at transcriptional or epigenetic dependencies may help restrict the reversible cell states that support dissemination, survival, and colonization. This may be particularly relevant in fusion-driven sarcomas, in which oncogenic fusions regulate chromatin state and enhancer landscapes. Second, the often immune-cold or myeloid-rich sarcoma TME suggests that immune checkpoint blockade alone is unlikely to be sufficient in most subtypes. More effective approaches may require combination strategies that enhance antigen presentation, target cancer-testis antigens such as NY-ESO-1 or MAGE when expressed, induce or mature tertiary lymphoid structures, or reprogram immunosuppressive macrophage-rich microenvironments [150]. Finally, the roles of dormancy and organ-specific metastatic niches indicate that preventing metastatic outgrowth may require targeting survival signals within the lung, bone, bone marrow, or other permissive microenvironments, rather than focusing exclusively on proliferating tumor cells [146].

Overall, ineffective therapies for sarcoma metastasis reflect limited mechanistic understanding of sarcoma dissemination, plasticity, immune escape, and metastatic niche adaptation. Translating these concepts into treatment will require subtype-specific biomarkers, longitudinal sampling, functional metastasis models, and clinical trials designed around sarcoma-specific biology rather than extrapolated carcinoma paradigms.

## Conclusion

Metastasis is a critical focus of oncology research in general and pediatric oncology in particular, as sarcomas account for about 15% of pediatric cancers and metastases drive poor outcome. Although much of the current framework derives from carcinoma models, sarcomas differ in cellular origin, genomic architecture, and dissemination modes. These differences suggest that sarcoma metastasis may not simply represent a variant of the epithelial paradigm, but a biologically distinct process.

Unlike carcinomas, sarcomas arise from mesenchymal tissues and frequently harbor single, dominant oncogenic events with low mutational burden. This genomic simplicity implies that metastatic competence may depend less on progressive genetic diversification and more on transcriptional plasticity and epigenetic reprogramming. In this context, pediatric fusion-driven sarcomas may represent tractable models for dissecting metastasis dependencies with high mechanistic resolution.

Evidence suggests that sarcoma cells may not require classical EMT programs to disseminate, but instead exploit intrinsic mesenchymal features including motility, cytoskeletal adaptability, and microenvironmental responsiveness. Metastatic progression may therefore depend less on epithelial–mesenchymal conversion and more on dynamic shifts between proliferative, migratory, stem-like, and immune-evasive states governed by tumor-intrinsic and microenvironmental cues.

Understanding how sarcoma cells interact with immune components, endothelial barriers, and organ-specific niches, particularly pulmonary and bone microenvironments, will be essential to identify actionable vulnerabilities. Advances in single-cell and spatial omics, lineage tracing, liquid biopsy, and improved experimental models offer opportunities to map metastatic trajectories and define whether metastatic competence is pre-existing or acquired during progression.

The core principles discussed here are not universal rules for every sarcoma subtype, but provide a framework for distinguishing sarcoma metastasis from carcinoma-derived paradigms. Reframing sarcomas not as imperfect carcinoma analogues, but as distinct models of mesenchymal, plasticity-driven, and microenvironmentally constrained metastasis, may help identify actionable vulnerabilities and improve outcomes for patients with disseminated disease.

## Data Availability

No datasets were generated or analysed during the current study.
